# Hydrogen sulfide in ageing, longevity and disease

**DOI:** 10.1042/BCJ20210517

**Published:** 2021-10-06

**Authors:** Stephen E. Wilkie, Gillian Borland, Roderick N. Carter, Nicholas M. Morton, Colin Selman

**Affiliations:** 1 Glasgow Ageing Research Network (GARNER), Institute of Biodiversity, Animal Health and Comparative Medicine, College of Medical, Veterinary and Life Sciences, University of Glasgow, Glasgow G12 8QQ, U.K.; 2 Molecular Metabolism Group, University/BHF Centre for Cardiovascular Sciences, Queens Medical Research Institute, University of Edinburgh, Edinburgh EH16 4TJ, U.K.

**Keywords:** aging, gasotransmitters, geroscience, hydrogen sulfide, longevity, progeria

## Abstract

Hydrogen sulfide (H_2_S) modulates many biological processes, including ageing. Initially considered a hazardous toxic gas, it is now recognised that H_2_S is produced endogenously across taxa and is a key mediator of processes that promote longevity and improve late-life health. In this review, we consider the key developments in our understanding of this gaseous signalling molecule in the context of health and disease, discuss potential mechanisms through which H_2_S can influence processes central to ageing and highlight the emergence of novel H_2_S-based therapeutics. We also consider the major challenges that may potentially hinder the development of such therapies.

## Biological generation of hydrogen sulfide (H_2_S)

### Endogenous production

Enzymatic production of H_2_S in mammalian tissues requires sulfur-containing amino acids (SAAs), specifically methionine and cysteine, as substrates [1,2]. Methionine cannot be synthesised *de novo* in mammals and must be consumed in the diet. In contrast, cysteine can be synthesised from methionine via conversion to homocysteine and is also consumed through diet. Homocysteine conversion into cysteine is referred to as the transsulfuration pathway (first described in the context of plant metabolism, in which cysteine is converted to homocysteine [3]). From cysteine, H_2_S is produced by two distinct canonical enzymatic pathways: directly through the activity of two pyridoxal-5′-phosphate (PLP)-dependent enzymes, cystathionine-gamma-lyase (CSE, or CGL) and cystathionine-beta-synthase (CBS), or indirectly through stepwise conversion into 3-mercaptopyruvate by l-cysteine:2-oxoglutarate aminotransferase (CAT) and then H_2_S by 3-mercaptopyruvate sulfurtransferase (MPST, or TUM1) [4]. The latter pathway is referred to the PLP-independent pathway as although CAT is PLP-dependent, MPST is not. These pathways are further distinguished by their sub-cellular localisation. CSE and CBS operate predominately within the cytosol, although both can translocate to the mitochondria under certain stress conditions [5]. For instance, CSE translocates to mitochondria during hypoxia, promoting H_2_S production within mitochondria and subsequently increasing ATP production [6]. Human MPST exists in two distinct isoforms, TUM1-Iso1 which is exclusively found within the cytosol and TUM1-Iso2, a splice variant encoding an additional 20 amino acid mitochondrial-targeting sequence [7]. The specific activity of mitochondrial MPST is two to three times higher than cytosolic MPST in rat liver [8]. While the pathways described above exclusively use the l-enantiomer of cysteine as a substrate, Kimura et al. [9] discovered a PLP-independent pathway for the production of H_2_S from d-cysteine (mainly in the cerebellum and kidney homogenates) through the action of MPST and d-amino acid oxidase in mitochondria and peroxisomes, respectively. While l-cysteine is the predominant, naturally occurring enantiomer of cysteine, common food processing practices rapidly racemise l-cysteine through heat and alkaline treatments, resulting in up to 44% conversion to d-cysteine [9]. The biologically relevant extent of this d-cysteine pathway remains unclear but presents an interesting alternative to the canonical mammalian production of H_2_S.

### Endogenous disposal

**Figure 1.**
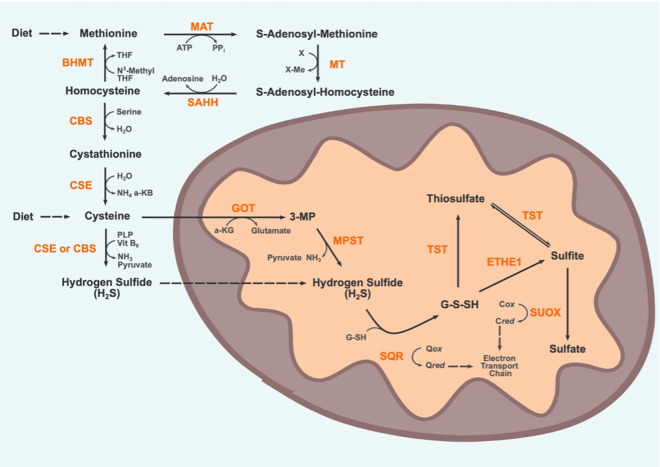
Substrate, intermediates and enzymes involved in the endogenous production and disposal of H_2_S. The blue region represents the cytosol, the orange region represents the matrix of a mitochondrion. The transsulfuration pathway cycles methionine into homocysteine first followed by enzymatic conversion of homocysteine into cysteine. From cysteine H_2_S is generated in the cytosol by CSE and CSE. H_2_S can also be generated within mitochondria by the action of MPST on 3-MP, a metabolite of cysteine. H_2_S can freely permeate membranes including the mitochondrial membranes. H_2_S disposal is carried out in mitochondria by several enzymes that comprise the sulfide oxidation unit (SOU). The precise mechanism of the SOU remains a subject of active research, the species and steps shown here represent just one proposed mechanism. Ultimately H_2_S is oxidised into sulfate which is subsequently excreted in the urine. MAT, Methionine adenosyl-transferase; ATP, Adenosine triphosphate; PPi, Inorganic pyrophosphate; X, Methyl group acceptor; MT, Methyltransferase; SAHH, S-adenosyl homocysteine hydrolase; BHMT, Betaine-Homocysteine S-methyltransferase; N3-Methyl THF, Trimethylglycine betaine; THF, Betaine; CBS, Cystathionine-β-synthase; CSE, Cystathionine-γ-lyase; NH_3_, Amine; a-KB, alpha ketobutyrate; PLP, pyridoxal 5′-phosphate; Vit B_6_, Vitamin B_6_; GOT, Glutamic-Oxaloacetic Transaminase; a-KG, alpha ketoglutarate; 3-MP, 3-Mercaptopyruvate; MPST, 3-Mercaptopyruvate Sulfurtransferase; SQR, Sulfur-Quinone oxidoreductase; Qox, Oxidised coenzyme Q; Qred, Reduced coenzyme Q; G-S-SH, Glutathione persulfide; ETHE1, Ethylmalonic encephalopathy 1 protein; TST, Thiosulfate Sulfurtransferase; SUOX, Sulfite Oxidase; Cox, Oxidised cytochrome C; Cred, Reduced cytochrome C.

Supraphysiological concentrations of H_2_S can be toxic, so efficient removal of H_2_S is performed by a suite of mitochondrial enzymes, collectively termed the sulfide oxidation unit (SOU) [10]. It has been shown that SOU actively catabolises H_2_S when intracellular concentrations exceed 10 nM in intact cells, with more restrictive thresholds observed in proximity to mitochondria [11]. However, determining a precise definition of supraphysiological H_2_S levels remains challenging due to limitations in detection methods and tissue and species specificity [12]. While the precise order of events and sulfur species involved in H_2_S oxidation are still unclear, the disposal of H_2_S consists of a series of oxidative reactions coupled to components of the electron transport chain within the mitochondria, ultimately yielding sulfate which is excreted in the urine. The first step in this pathway is the oxidation of H_2_S by the flavoprotein sulfur:quinone oxidoreductase (SQR) [13] catalytic cycle whereby the flavin cofactor is cyclically reduced by H_2_S and oxidised by ubiquinone, with coenzyme Q acting as an electron acceptor. It is through coenzyme Q that H_2_S metabolism is coupled to ATP generation by oxidative phosphorylation, making H_2_S a rare example of an inorganic compound capable of fuelling mammalian oxidative phosphorylation [14]. The product of this enzymatic cycle is the generation of SQR-persulfide intermediates, which are transferred primarily to glutathione (GSH) in human tissues, generating glutathione persulfide (GSSH) [15]. SQR is also capable of catabolising H_2_S to produce thiosulfate from sulfite, although low tissue levels of sulfite makes it unclear whether this reaction accounts for a substantial proportion of physiological SQR activity in mammals, despite orders of magnitude greater reactivity with persulfidated SQR compared with GSH [16,17]. GSSH is oxidised by ethylmalonic encephalopathy 1 (ETHE1) or thiosulfate sulfurtransferase (TST) to form sulfite or thiosulfate, respectively. ETHE1 is a sulfur dioxygenase, consuming O_2_ and water as substrates to oxidise H_2_S [18]. TST may then reversibly convert thiosulfate to sulfite which is irreversibly oxidised into sulfate by sulfite oxidase (SUOX). Both sulfate and thiosulfate are removed via the circulatory system and then ultimately excreted in the urine [19]. Overall, disposal of 1 H_2_S molecule requires the consumption of 0.75 O_2_ molecules; 0.5 by ETHE1 and 0.25 by Complex III [10]. The enzymatic generation of H_2_S from SAAs and its subsequent removal are detailed in Figure 1. Of note, as mature red blood cells (RBCs) typically lack mitochondria, they utilise a methaemoglobin pathway for the disposal of H_2_S by conversion of H_2_S into thiosulfate and polysulfides [20]. It remains an open question as to whether the methaemoglobin pathway for H_2_S oxidation found within RBCs is utilised in other tissues in mammals.

### Bacterial production

Putrefaction of decaying organic matter in anaerobic conditions results in the production of H_2_S [21]. This is due to the action of a wide range of sulfate-reducing bacteria (SRB) which utilise sulfate as a terminal electron acceptor for respiration, with the concomitant production of H_2_S [22]. There is a wide range of such SRB within the microbiome of the human colon, primarily of the genus *Desulfovibrio* in the class d-Proteobacteria [23]. Endogenous production of H_2_S in bacteria is catalysed by orthologs of CSE, CBS, and MPST [24]. The interactions between groups of bacteria are complex and poorly understood. SRB use a wide range of substrates including lactate, hydrogen, short-chain fatty acids, and amino acids, which places them in direct competition with other bacterial species such as hydrogenotrophic bacteria, methanogens, and acetogens. However, SRB appears to dominate the use of hydrogen in the microbiome as they are capable of catabolizing hydrogen at concentrations far lower than other hydrogenotrophic species [25]. It is currently difficult to directly measure the proportion of H_2_S produced by bacteria compared with endogenous enzymatic production in tissues. Germ-free mice have 50% less measurable H_2_S in faecal samples compared with control mice and are capable of altering SRB-activity to compensate for the impairment in enzymatic H_2_S production following a PLP-deficient diet [26]. H_2_S gas produced by the microbiome in the gut can enter proximal human tissues or the bloodstream [27]. For instance, high levels of SRB-derived H_2_S inhibits butyrate oxidation, the major source of energy production in intestinal colonocytes [28]. Furthermore, there is evidence that bacterial-derived H_2_S can reduce arterial blood pressure in rats [29], and contradictory evidence points to either a therapeutic or causative role of H_2_S in inflammatory bowel disease and colorectal cancer [30]. Additionally, there is potential for diet to influence the relative abundance of SRB, as diet has been shown to modify microbiome composition in general [31]. However, no significant effect of short-term adoption of diets either enriched for or deficient in SAAs was observed on relative SRB populations in stool samples from healthy human volunteers [32]; future studies employing longer-term dietary interventions and greater statistical power are required to further clarify this question. Finally, it has been proposed that bacterial production of H_2_S protects the bacteria against oxidative stress and may contribute to antibacterial resistance [33]. For example, Shatalin et al. [33] developed novel small molecule inhibitors of bacterial CSE and found these inhibitors improved antibiotic potency against *Staphylococcus aureus* and *Pseudomonas aeruginosa in vitro* and in mice, supporting the theory that endogenous production of H_2_S in bacteria might contribute to antibacterial resistance. We believe research using germ-free mice is one approach that may help provide more information regarding the relevance of SRB-derived H_2_S in whole-animal metabolism and physiology.

## Signalling modalities of H_2_S

### Post-translational modification (persulfidation)

**Figure 2.**
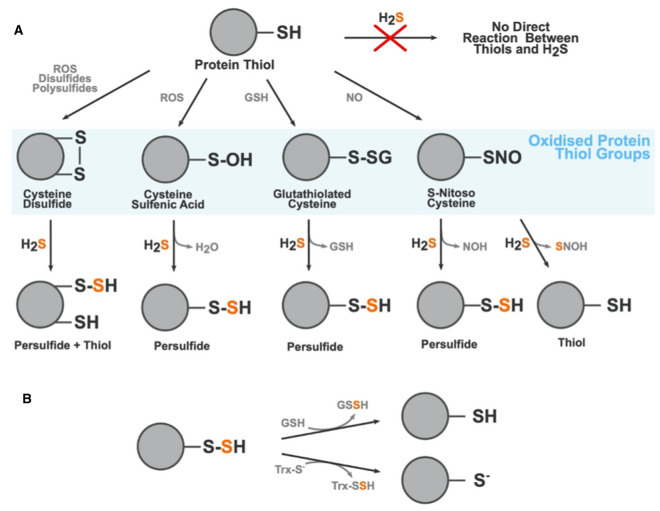
Formation of protein persulfides by H2S. (**A**) Modification of cysteine residues by H_2_S. H_2_S cannot directly modify thiol groups (i.e. cysteine residues). The thiol group must first be must first be oxidised into a disulfide (disulfide bond formation), sulfenic acid (S-Sulfenylation), glutathiolated cysteine (S-Glutathiolation), or a S-Nitroso Cysteine (S-Nitrosation). From these oxidised thiol groups H_2_S can react to form persilfides, thiols, and a variety of by-products dependent on the type of oxidised thiol it is reacting with. The sulfur atom from the H_2_S molecule is highlighted in orange to show where in the product it incorporates. (**B**) Persulfidation is a reversible post-translational modification and can be readily removed by the action of glutathione and thioredoxin. ROS, Reactive oxygen species; GSH, Glutathione; NO, Nitric oxide; NOH, Nitroxyl; SNOH, Thionitrous acid; GSSH, Glutathione persulfide; Trx-S^−^, Thioredoxin.

Protein modification by H_2_S is a reversible post-translational modification that can occur on any cysteine residue. Overall, the thiol group (R-SH) present in cysteine is indirectly changed to a persulfide group (R-S-SH), a process known as persulfidation or sulfhydration. The thiol group must first be oxidised to form thiol derivatives such as sulfenic acid (R-SOH), a disulfide (R-S-S-R), or S-nitrosothiol (R-SNO), which can then react with H_2_S to create a persulfidated protein residue. A schematic showing the various thiol derivatives H_2_S can react with and their subsequent products are shown in Figure 2, adapted from [34]. Persulfides are highly reactive, with a neucleophilic terminal sulfur atom and an electrophilic inner sulfane sulfur atom [35]. Persulfidation of cysteine residues causes conformational changes in protein structure that alter protein activity such as the regulation of Kelch-like ECH-associated protein 1 (Keap1), which has well-characterised conformational regulation through alterations of cysteine residues [36,37]. Keap1 is the major inhibitor of the nuclear factor erythroid 2-related factor 2 (NRF2)-mediated antioxidant response mechanism. *In vitro* approaches have shown alteration of cysteine residues on Keap1 following exposure to H_2_S leading to inactivation of KEAP1, but currently, there is no agreement on the precise residue(s) persulfidated in this process [36,37]. Another established persulfidation target is the Kir6.1 subunit of K_ATP_ channels which confers cardioprotective effects when activated by H_2_S [38]. An extensive review of the chemistry of persulfides, their molecular targets, and role in various tissues and diseases was compiled by Filipovic and colleagues in 2017 [39]. Persulfides decay under biologically relevant conditions, which poses a challenge in the identification, measurement, and characterisation of persulfidated species in biological contexts. The half-life of Cys-S-SH is ∼35 min at 37°C [40]. Spontaneous removal of persulfides is caused by a disproportionation reaction between two persulfides to form many sulfur-containing species including: elemental sulfur, thiols, polysulfanes, and/or H_2_S [40–42]. Additional processes that can break down persulfides include homolysis by heat or light and enzymatic removal by the thioredoxin system. Given these constraints, it is difficult to achieve a full understanding of the dynamics of protein persulfidation as most methods take a ‘snap-shot' of global persulfidation at one time. Despite these limitations, our understanding of the extent of protein modification by persulfidation, collectively termed the persulfidome, is growing. In *Arabidopsis thaliana*, for example, 5% of the proteome was found to be persulfidated using modified tag-switching protocol that employed methylsufonylbenzothiazole (MSBT) to block both thiol and persulfide groups within the sample [43]. This was then followed by the addition of CN-biotin which does not react with MSBT adducts of thiol origin and therefore allows for streptavidin-based pull-down of persulfidated proteins [43]. Additionally, proteomic studies in wild-type mice have found 10–25% of hepatic proteins to be persulfidated under physiological homeostasis [44]. Comprehensive work by Zivanovic et al. [45] showed that a high degree of hepatic protein persulfidation is associated with an extended lifespan, augmented by dietary restriction (DR), and diminished with age; these trends were conserved across model organisms. Bithi et al. [46] described tissue-specific changes in the persulfidome of mice exposed to 50% DR and in mice homozygous null for CSE. As persulfidation can in principle occur on any cysteine residue, and is a highly dynamic, reversible post-translational modification, there is enormous scope for H_2_S to modify proteins in a variety of biological settings.

### Binding with metal centres

H_2_S is capable of binding to multiple metal ions, the most direct signalling modality in its repertoire. Upon binding, the coordination, charge, and oxidation states of the metal ion may be altered [47]. Such reactions become biologically relevant in the context of metalloproteins which contain metal centres in their quaternary structure. Metalloproteins represent a significant percentage of all mammalian proteins, with recent estimates suggesting that approximately 6600 human proteins are metalloproteins [48], or approximately a third of all protein products. H_2_S reaction with haemoproteins is well established, particularly with ferric haemoglobins but also metmyoglobins, methaemoglobins, and peroxidases [49]. In fact, the much-discussed toxicity of H_2_S is a result of its highly efficient inhibition of cytochrome c oxidase (COX, also known as Complex IV in the electron transport chain). COX is a dimer formed of subunits that include two heme, two copper, one magnesium, and one zinc centre [50]. Inhibition of COX by H_2_S occurs in a biphasic manner under a complex series of reactions with the haem and copper centres, forming intermediates that are currently unresolved [51]. Furthermore, H_2_S inhibits angiotensin-converting enzyme by binding to a zinc atom at the active site, with dose-dependent inhibition of this enzyme demonstrated in protein lysates from human endothelial cells [52]. Interestingly, binding with haem centres in haemoglobin may be the major H_2_S clearance pathway in RBCs [20]. It is established that RBCs do produce endogenous H_2_S, primarily through MPST, but as they lack mitochondria in most mammals they do not possess the canonical clearance mechanisms (see section Endogenous disposal). Unchecked, H_2_S production in the trillions of RBCs within the circulation would inevitably result in a lethal build-up of H_2_S. However, it appears that a cycle of reactions between H_2_S species and haemoglobin results in the oxidation of H_2_S into reactive sulfur species (RSS) such as thiosulfate and hydropolysulfides [20]. A similar process appears to occur between H_2_S and myoglobin in cardiac and skeletal muscle [53].

### Interaction with other gasotransmitters

**Figure 3.**
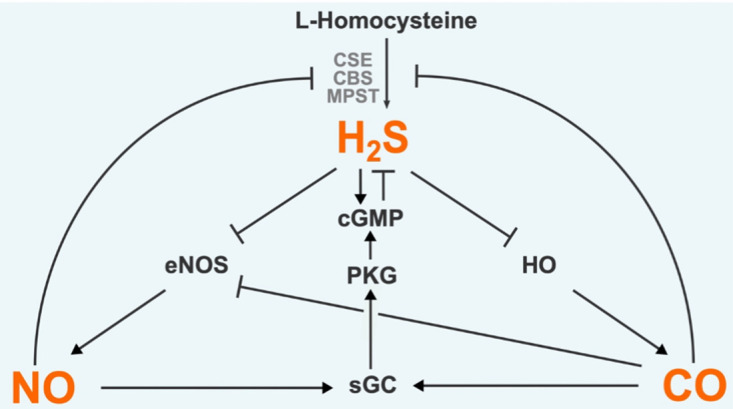
Known interactions between H_2_S, CO, and NO signalling pathways. Each gasotransmitter is capable of regulating the other two. Pointed arrows represent a stimulatory effect. Flat-headed arrows indicate an inhibitory effect. H_2_S, Hydrogen sulfide; NO, Nitric oxide; CO, Carbon monoxide; CBS, Cystathionine-β-synthase; CSE, Cystathionine-γ-lyase; MPST, 3-Mercaptopyruvate sulfurtransferase; eNOS, Endothelial NO synthase; HO, Heme oxygenase; sGC, Soluble guanylyl cyclase; PKG, Protein kinase G; cGMP, Cyclic guanosine monophosphate.

H_2_S is not alone as a gasotransmitter. Other compounds with similar properties are carbon monoxide (CO) and nitric oxide (NO). These gases are also toxic at high concentrations, are produced endogenously, and can freely permeate plasma membranes to exert biological effects. All three gasotransmitters are highly reactive producing various metabolites that are collectively termed RSS, reactive oxygen species (ROS), and reactive nitrogen species (RNS). It has become clear that these reactive chemical species can react with metabolites and derivatives of the other gasotransmitter molecules to form a densely interconnected web of products sometimes collectively termed the reactive species interactome. For instance; H_2_S, NO and their derivatives react to form a family of nitrothiol compounds, resulting in modulation of signalling pathways [54]. Furthermore, each gasotransmitter is capable of regulating the production of the other two gasotransmitters (Figure 3). H_2_S stimulates NO production through transcriptional, translational, and post-translational interventions in the NO synthesis pathway, with reports of both elevation and suppression of NO production [55]. The mechanism by which H_2_S elevates CO production is still an area of active research but appears to involve activation of the Nrf2-mediated response (see section Post-translational modification (persulfidation)) up-regulating heme oxygenase isoforms which generate CO [56]. These chemical species and intermediates are highly dynamic which makes measuring and understanding the exact processes involved in H_2_S-NO-CO cross-talk challenging. What is clear is that such cross-talk is an important signalling modality across a diverse range of organisms, influencing plant growth and ripening for example [57,58]. In mammals, the dynamics and functions of H_2_S-NO-CO cross-talk are best understood in the cardiovascular system where they exert control over inflammation, angiogenesis, vasodilation, and protection from ischaemia-reperfusion injury (IRI) [59,60]. An interesting case study in the complexity of gasotransmitter cross-talk is demonstrated by the regulation of the activity of soluble guanylate cyclase (sGC), a hemeprotein. Overall, the three gasotransmitters all increase sGC activity but the biochemistry involved in this outcome are distinct. NO is an exceptionally strong activator of sGC, augmenting sGC activity over 100-fold [61]; in contrast, CO is a far weaker activator of sGC [62]. Due to this disparity in potency and binding strength, NO and CO compete for dominance in their interaction with sGC: when NO concentrations are low CO is the predominant activator of sGC; but when NO concentrations are high CO actually inhibits NO-induced elevation of sGC activity [60]. Distinct from this, H_2_S does not directly activate sGC but instead has been shown to reduce the heme moiety from Fe^3+^ to Fe^2+^ in human recombinant sGC. CO and NO only interact with Fe^2+^ sGC, thus H_2_S facilitates the activity of the other two gases by increasing the available pool of Fe^2+^ sGC [63]. Thus, all three gases work to elevate sGC activity but there is considerable nuance in how this is achieved. The remainder of this review will focus on the effects of just one gasotransmitter, H_2_S, in health, disease, and ageing. However, in light of the intricate and overlapping effects of all three gasotransmitters, we must be mindful of the possibility that any effects attributed to H_2_S may in reality belong to the unity of all three gasotransmitters.

## H_2_s and ageing

### Role of H_2_S in normative ageing

Exploration of the processes that underlie ageing is most easily understood under the guidance of the hallmarks of ageing [64], a landmark review that proposed nine discrete categories of biological processes that are conserved in organismal ageing. A recent review by Perridon et al. [4] considered the impact of H_2_S on each of these hallmarks in turn and collected evidence showing direct, H_2_S-mediated protection from all ageing hallmarks except for telomere attrition, for which no studies had been published. This review will not aim to repeat the work previously published but instead assess subsequent publications concerning the effect of H_2_S on specific tissue ageing. Whilst it is probable that the dynamics of H_2_S production and activity are altered throughout age in most tissues of the body, recent papers have focussed on a few select organ systems including the heart, brain, and kidneys.

#### Cardiovascular ageing

The typical progression of cardiovascular ageing is initiated by endothelial dysfunction, leading to vascular dysfunction, increased severity of atherosclerosis, and subsequently cardiovascular diseases (CVDs) including stroke, hypertension, and coronary heart disease [65]. Key molecular mechanisms that drive this pathological progression are under the influence of H_2_S including: signalling through Nrf2, SIRT1, and AMPK/mTOR; activation of potassium channels; and regulation of mitochondrial biogenesis by PGC-1a [66]. Furthermore, exposing cells and mice to H_2_S can ameliorate age-associated vascular ageing [67]. Treatment of cultured endothelial cells with nicotinamide mononucleotide (NMN, an NAD^+^-elevating supplement) improves vascular remodelling in response to ischaemic injury and enhances endurance and capillary density in old mice, effects that are augmented by co-treatment with H_2_S-donating compounds [67]. The augmentation of vascular health by NAD^+^ and H_2_S boosting treatment is proposed to be due to the convergence of these signalling pathways through SIRT1. However, the same authors also reported that treatment with H_2_S in isolation enhanced basal mitochondrial respiration levels in HUVEC cultures, an effect not seen when using NMN [67]. This indicates that H_2_S has protective effects independent of NAD. Other evidence for a protective role for H_2_S in cardiac cell culture models include improved glucose utilisation, improved metabolic efficiency of glycolysis and the citric acid cycle, and protection against induced cardiomyocyte hypertrophy [68]. Furthermore, CSE expression and H_2_S production were found to be reduced in a model of aged primary rat cardiomyocytes [69]. Treatment of these cells with sodium hydrosulfide (NaHS, a H_2_S-donating compound) improved cardioprotection in response to ischaemia-reperfusion events via inhibition of mitochondrial permeability transition pore opening and improved mitochondrial membrane potential [69]. Peleli et al. [70] used a mouse model with knock-out (KO) of MPST, one of the three enzymatic producers of H_2_S (see section Endogenous production), to study the effect on sulfur-containing chemical species. In these MPST KO mice there was no significant effect on H_2_S, polysulfides, or sulfane sulfur level in heart tissue, nor did it affect blood pressure or vascular reactivity relative to wild-type controls, but did elevate several cardiac ROS markers [70]. However, while some positive cardioprotective phenotypes were observed in these mice at 2–3 months of age (including protection from IRI), deleterious phenotypes (including hypertension, cardiac hypertrophy, and reduced myocardial nitric oxide production) were reported at 18 months of age [70]. The authors suggest that the cardioprotective effects in young mice could be explained by increased cardiac ROS levels providing a pre-conditioning against IRI, whereas at old age it appears that ablation of MPST is deleterious to heart function. This study is the first to investigate the cardiovascular phenotype in MPST KO mice and further studies should aim to extend understanding in the role and pathophysiology of MPST in the onset of age-related heart disease.

#### Neurological ageing

As neuromodulation was the first functional role described for endogenous H_2_S in humans [71], it is unsurprising that H_2_S has been implicated as a key player in brain ageing. One conduit for multiple neuropathological processes is the receptor for advanced glycation end-products (RAGE). RAGE is among several receptors that bind to advanced glycation end-products, proteins and lipids that have been modified by reaction with sugar molecules in a non-enzymatic manner that accumulate in tissue with age, including the brain [72]. It should be noted that while the transmembrane forms of RAGE are implicated in neurotoxic signalling, soluble forms of RAGE have instead been shown to confer neuroprotective effects, in part due to inhibition of membrane-associated RAGE [73]. RAGE also binds to beta-amyloid, engendering deleterious effects and, as such, has drawn interest as a potential target in the treatment of Alzheimer's disease [74]. Treatment with exogenous H_2_S in cells has been shown to inhibit stabilisation of membrane-associated RAGE dimers and the modality for this inhibition was direct persulfidation of a cysteine residue on RAGE [75]. Beyond RAGE signalling, other ageing processes are subject to H_2_S regulation in neural cell systems. In a cell culture model of hyperglycaemia-induced hippocampal senescence, treatment of cells with a H_2_S donor resulted in a reduction in senescence markers and improved autophagic flux in a SIRT1-dependent manner [76]. H_2_S also influences synaptic plasticity, as shown by Abe and Kimura's work on H_2_S-facilitated long-term potentiation (LTP) [71]. Thus, stimulation of *N*-methyl-d-aspartic acid (NMDA) receptors in active rat hippocampal synapses was augmented by AdoMet, a CBS-activating compound [71]. More recently, Lu et al. [77] screened a group of aged mice on cognitive ability and showed CBS protein levels were significantly lower in mice with impaired cognition and that the cognitive impairment in these mice was rescued following administration of a H_2_S donor (NaHS). These effects were associated with altered sensitivity of metabotropic glutamate receptors to local calcium levels [77], likely due to H_2_S modulation of neuronal calcium homeostasis [78]. Similarly, the ability of rats to learn an adaptive associative response to fear conditioning was dependent on endogenous H_2_S production by CBS [79]. When CBS was inhibited by hydroxylamine or amino-oxyacetate, amygdalar and hippocampal H_2_S levels were reduced, NMDA-receptor mediated LTP was significantly impaired, and fear conditioning responses were dampened. All these effects were rescued by the application of H_2_S donor compounds, even in the presence of CBS inhibitors, indicating that the loss of H_2_S production is what mediates these effects. In agreement, a similar reduction in fear conditioning-stimulated LTP due to reduced tissue H_2_S production and reversal of this effect by application of a sulfide donor was observed in synaptic plasticity in aged rats [80]. H_2_S also modulates the biological response to ischaemic stroke, which accounts for over 80% of all strokes [81]. Both endogenous and exogenous sources of H_2_S confer neuroprotective effects at low doses and deleterious effects at higher doses. For instance, H_2_S production via CBS is greatly elevated following stroke and inhibitors of CBS activity reduced infarct volume in rat models of stroke, whereas administration of H_2_S-donating compounds increased infarct volume [82]. However, elevated H_2_S activity ameliorated deleterious pro-inflammatory response co-ordinated by microglia, a major contributor to the cerebral IRI pathology. Inhalation of a low dose of H_2_S for 3 h immediately after induced cerebral IRI in rats resulted in suppression of this inflammation response through protein kinase C-dependent reduction in aquaporin 4 protein expression, resulting in a reduction in ischaemia infarct size and improved neurobehavioral outcomes [83].

#### Renal ageing

H_2_S production in the kidney is driven by CSE and CBS activity with expression of these enzymes concentrated particularly within the proximal tubule [84]. As H_2_S production through these enzymes is part of the transsulfuration pathway there is overlap with homocysteine metabolism which is associated with mortality in late-stage kidney disease [85]. Given the kidney's role in filtering blood content, it is unsurprising that they are sensitive to nutritional intake. Various studies demonstrated a link between diet composition and renal ageing, with amino acid content emerging as a key driver. Dietary restriction (DR) is the most well-characterised intervention for improving health and lifespan (see section H2S in dietary restriction) and typically involves a reduction in gross calories consumed within a set period [86]. However, recent studies have highlighted a specific requirement for restriction of essential amino acids (EAA) in DR protocols for renal protective effects to occur [87]. In a study by Yoshida et al. [88] mice were placed under ‘simple DR’ (40% reduction in calorie intake) and DR with supplementation of EAAs (DR + EAA) or non-EAA (DR + NEAA). They found that while DR and DR + NEAAs groups displayed extended lifespan and protection from tubulointerstitial lesions, these effects were lost in groups subjected to DR + EAA supplementation. More specifically, they found that excluding methionine from the EAA supplementation was sufficient to restore DR-induced benefits on longevity, kidney function and oxidative stress, and was correlated with an increase in tissue H_2_S levels and increased CSE gene expression. Wang et al. similarly found that methionine restriction alone was sufficient to extend lifespan and improve markers of renal ageing in mice. Their mechanistic investigations suggest that AMPK-dependent H_2_S signalling protected kidney tissue from the onset of senescence [89]. Additionally, various histological and functional markers of renal ageing were described in both male and female marmosets between ∼3 and 16 years of age, with these changes correlating with an age-associated reduction in CBS protein levels across both sexes, although a significant age-associated reduction in H_2_S production was observed only in male marmosets [90]. Another major consequence of renal ageing is acute kidney injury (AKI), which is driven in part by IRI [91]. A single incidence of AKI has profound implications for mortality; hospital patients with AKI commonly have 30–40% mortality rates and as high as 60% for AKI patients admitted to intensive care units [92]. Renal IRI can be ameliorated by the action of H_2_S and NO signalling which improve blood flow by causing local vasodilation, inhibiting inflammatory cytokines, and reducing ROS production [93].

### H_2_s in lifespan extension

Ageing is plastic and modifiable by a variety of environmental, genetic, and pharmaceutical interventions [86]. This section will consider established lifespan extension interventions and assess the potential mechanistic role of H_2_S in their modulation of biological ageing.

#### H_2_s in dietary restriction

DR is an umbrella term for a panel of interventions that have been known to consistently improve longevity across taxa for more than 100 years [94–96]. The conservation of this response suggests an evolutionary origin of longevity through DR, best understood through the framework of the disposable soma, mutation accumulation, and antagonistic pleiotropy theories of ageing, among others [97,98]. DR typically confers significant health benefits, and improves late-life health by reducing the incidence and/or trajectory of many age-related pathologies, including cognitive decline, metabolic syndrome, CVD and many cancers [94,99]. Many of these health benefits are also observed in non-human primates exposed to life-long DR [99]. However, cognitive defects under DR have been reported in rats and atrophy of grey matter volume in DR fed primates [100,101]. Critically, many of the positive health benefits found in model organisms under DR are replicated in humans under DR protocols that carefully supply 100% of essential daily nutrients, but the impact on lifespan is currently unknown [94,95]. The application of DR as a preventive therapeutic tool in humans is promising [102] but remains a challenge, largely due to the difficulty in avoiding accidental malnutrition. Additionally, DR in humans has several reported drawbacks including infertility, sarcopenia, osteoporosis, and reduced immunity [103]. As such, the challenges of applying DR in the wider human population are prohibitive and we may be better served by gaining an understanding of the mechanisms that underlie DR and designing therapeutics targeting them more selectively.

Our understanding of the mechanisms that underpin the effect of DR on lifespan remain imprecise despite decades of investigations. What is certain is a major contribution to DR-induced longevity is from reduced nutrient signalling and improved insulin sensitivity through modulation of signalling pathways including mTOR, insulin/insulin-like signalling (IIS), and NAD metabolism. Murine models with compromised TOR or IIS signalling molecules (such as global loss of ribosomal S6 protein kinase 1 or insulin receptor substrate 1, respectively) showed marked increases in lifespan and a delay in age-related physiological decline [104,105]. Several studies identified H_2_S as a potentially conserved mechanism underlying DR-induced longevity and healthspan improvements. In a series of seminal papers led by Dr James Mitchell, the positive effects of multiple DR regimes were dependent on elevated H_2_S production in yeast, worms, fruit flies, and mice [106–110]. It is also clear that the effects attributed to DR can largely be recapitulated by the removal of specific dietary components from the diet, even if total calorie intake is maintained [111]. Such interventions include restriction of total protein or tryptophan intake, but perhaps the best studied is methionine restriction, which appears to be closely tied to the transsulfuration pathway and H_2_S homeostasis [94]. Life-long methionine restriction in mice protected against renal senescence and elevated endogenous H_2_S production, with complementary *in vitro* assays indicating a mechanistic role for H_2_S in this protection [89]. Given that the SAAs (methionine and cysteine) are the canonical sources for endogenous *de novo* H_2_S production, it is perhaps unsurprising that restriction of methionine modulates H_2_S production. However, it is counterintuitive that restriction of the dietary source for *de novo* H_2_S synthesis ultimately results in elevation of H_2_S levels; a conundrum that has several possible solutions but no concrete answer to date [107]. One resolution to this apparent contradiction is that DR reduces hypothalamic–pituitary signalling, which functions partly through the inhibition of H_2_S production by growth hormone and thyroid hormone at the transcriptional and protein levels, respectively [112]. As such, DR-mediated reduction in growth and thyroid hormone release may reduce inhibition of H_2_S production enzymes. One alternative explanation for the observation that reduced calorie intake elevates H_2_S levels despite reduced pools of SAAs is that elevation of autophagic processes under nutrient-limiting conditions generates the substrate pool for H_2_S biogenesis. DR and fasting interventions have been shown to elevate autophagy processes across tissues in mice and humans [113]. Indeed, induction of H_2_S biogenesis under DNA damage stress has been demonstrated to be a autophagy-dependent response *in vitro* [114], and cysteine pools are maintained through autophagic processes in pancreatic cancer [115]. Methionine has also been shown to indirectly inhibit the induction of autophagy by elevating S-adenosylmethioine (SAM) levels, which in turn promotes methylation of protein phosphatase 2A, leading to autophagy inhibition [116]. Together, these studies support the premise that elevated autophagy replenishes the cellular cysteine pool, allowing for the generation of H_2_S under nutrient-limiting conditions. More studies that directly measure H_2_S levels under such conditions are required to definitively support this.

#### H_2_s in dwarf mouse models

Beyond dietary interventions, various mutations in model organisms confer significant longevity benefits. In fact, the Ames dwarf mouse has the longest extension in lifespan achieved by genetic, dietary, or pharmaceutical intervention with mean and maximal lifespan increase in over 45% in both sexes [117]. The dwarf mouse models have genetic disruption of anterior pituitary gland function either through mutations in transcription factors like Pit1 and Prop1 (as in the Snell and Ames dwarf mice models, respectively) or in growth hormone signalling receptors such as growth hormone receptor and growth hormone-releasing hormone receptor, both of which result in long-lived dwarf mice [117–119]. There have been relatively few studies that link the reduced pituitary signalling phenotype to the action of H_2_S, with the notable exception of Hine et al. [112] who showed that both the Snell and Ames dwarf models had up-regulation of H_2_S production pathways. This is in part due to ablation of the transcriptional regulation of CSE and CBS expression by thyroid hormone signalling and through substrate availability control by autophagic processes, respectively, in dwarf mice [112]. This correlates well with previous research that used labelled metabolites to demonstrate an increase in the flux of methionine through the transsulfuration pathway in Ames mice [120]. These studies unveiled a rerouting of metabolism through transsulfuration in the liver, brain, and kidneys of the mice with a concomitant, but non-significant, increase in hepatic CSE gene expression compared with wild-type controls [120]. Hepatic CSE specific activity is also elevated in Ames mice [121]. The expected result of this altered metabolism is that the Ames mice will have an elevated pool of cysteine from which H_2_S can be generated, which may contribute to the findings of Hine et al. [112] that these mice have improved H_2_S production capacity. Interestingly, while restriction of dietary methionine extended lifespan and increased hepatic H_2_S levels in many models, the Ames models showed no increased lifespan on a methionine-restricted diet [122]. H_2_S levels have not been measured in Ames mice under methionine-restricted conditions, however, Brown-Borg et al. [123] showed that much of the rerouting of metabolic processes through transsulfuration observed in Ames mice was unaffected by methionine restriction. This was opposed to the expected up-regulation of transsulfuration as seen in wild-type animals on methionine restriction [123]. From this, we could infer that intact growth hormone signalling is essential for ‘sensing’ dietary amino acid abundance and plays an important role in coordinating altered metabolism in response to differential methionine abundance. Further work is required to assess if H_2_S plays a role in this proposed mechanism for growth hormone regulation of methionine metabolism as well as in the extraordinary lifespan extension of growth hormone mutant mice.

#### H_2_s in longevity through pharmaceutical intervention

Longevity is plastic in response to a variety of pharmaceutical interventions, and chief among these are inhibitors of nutrient-sensing pathways such as Rapamycin (targets mTOR signalling), and the anti-diabetic drugs Metformin (targets AMPK signalling) and Acarbose (targets IIS signalling) [94]. H_2_S signalling overlaps with all of these mechanisms.

##### Rapamycin and mTOR signalling

Within the context of mTOR signalling, H_2_S can be either stimulatory or inhibitory, as recently reviewed [124]. This is counterintuitive as both H_2_S and Rapamycin were implicated as pro-longevity molecules and therefore we might anticipate they would both act upon the mTOR pathway in a similar manner, i.e. suppression of mTOR activity. This is the case in some instances, such as a study in brain tissue from diabetic mice where treatment with a H_2_S donor reduced protein synthesis by inhibiting mTOR signalling and increasing autophagic processes [125]. Furthermore, exogenously increased H_2_S concentration induces autophagy in cells and is associated with inhibition of TOR activity [126,127]. However, contradictory studies showed an anti-autophagic role for H_2_S via mTOR signalling with myriad effects ranging from rescuing high-fat diet-induced liver disease, protecting against diabetic myopathy, stimulating angiogenesis, and stimulating osteoclastogenesis [128–131]. Along with conflicting results in mTOR signalling, we lack a full appreciation of the effect of Rapamycin on H_2_S production pathways. To date only one study has investigated this, using Rapamycin in *Saccharomyces cerevisiae* and human cells [132]. The authors found that Rapamycin inhibited H_2_S production through the depression of CSE and CBS gene transcription in both cell models, indicating a conserved role of Rapamycin in regulating H_2_S generation [132]. More work is required to test how conserved this response to Rapamycin treatment is across tissues and species. There also remains a lack of studies that combine Rapamycin and H_2_S donors. Such approaches offer an additional understanding of how these compounds co-interact with mTOR signalling. One example of such an approach used a human hepatocellular carcinoma cell line and treatment with Rapamycin and a H_2_S-donor separately or in combination [133]. Wang et al. also found that both treatments inhibited mTOR signalling and stimulated anti-tumour autophagic and pro-apoptotic pathways and were additive when used in combination. The sum of work performed by researchers has confirmed the theory that longevity through Rapamycin inhibition of mTOR is subject to regulation by H_2_S. However, further studies are required to dissect out the precise conditions where H_2_S modulates mTOR in alignment with Rapamycin, in opposition, or whether there is a more nuanced interaction between these molecules.

##### Metformin and AMPK signalling

Metformin is another putative lifespan-extending drug that interacts with H_2_S signalling. Metformin's mechanism of action remains only partially resolved, but appears to operate largely through activation of AMPK (which in turn inhibits mTOR and IIS signalling pathways) [134]. Early studies showed that there was a correlational link between metformin treatment in mice and the elevation of H_2_S levels in the brain, heart, kidney, and liver tissues [135]. Following this discovery, the role of H_2_S in the pharmacological activity of AMPK signalling and metformin treatment was studied in earnest and this body of work was collected in a 2017 review [136]. How metformin increases H_2_S levels is becoming increasingly apparent and appears related to the ability of metformin to remodel DNA methylation patterns [137]. Work by Ma et al. [138] showed that a high methionine diet (methionine forming 2% of diet) resulted in the elevation of plasma homocysteine levels and a reduction in plasma H_2_S levels, effects that were rescued by metformin treatment. Complementary cell culture assays suggest that metformin treatment removes homocysteine-stimulated hypermethylation of the *CSE* promotor region, resulting in greater mRNA and protein expression of CSE and elevation of H_2_S production [138]. Similarly, a metabolomics study in rats found that metformin treatment ameliorated oxidative liver damage caused by exposure to bisphenol A through elevation of CSE and CBS levels [139]. Our emerging understanding of the transcriptional control of H_2_S producing genes presents a clear connection between metformin and H_2_S production. However, as the modes of action of metformin remain only partially understood, more work is required to fully understand the interplay between H_2_S, AMPK signalling, and metformin.

##### Acarbose and IIS signalling

Acarbose inhibits carbohydrate digestion and glucose absorption and is known to extend maximum lifespan in male and female mice, but only extends median lifespan in males [140]. There is currently a scarcity of studies interrogating the interaction of Acarbose with H_2_S. This presents a potentially fruitful area of novel research as H_2_S is already known to modulate insulin signalling and whole-animal glucose metabolism across tissues, cellular processes that appear intimately linked with longevity [141]. As with other signalling pathways, the effects of H_2_S are complex, with independent studies reporting either protective or deleterious effects [142]. The endogenous production of H_2_S in adipose cells was first described by Feng et al. [143] who showed that elevated CSE expression and H_2_S production was correlated with insulin resistance in rats, suggestive of a deleterious diabetic phenotype associated with H_2_S expression in adipocytes. Similar results were found in a hepatocyte cell line and primary mouse hepatocytes which showed that supraphysiological levels of H_2_S, either through H_2_S donor compounds or adenovirus-induced overexpression of CSE, negatively impacted glucose uptake and storage as glycogen [144]. These effects were attributed in part to inhibition of both the AMPK and IIS signalling pathways [144]. Finally, pancreatic beta-cells under chronic exogenous H_2_S treatment exhibited suppression of insulin secretion and were protected against oxidative stress-induced apoptosis via elevated glutathione content and reduced ROS [145]. The authors suggest that this cytoprotection may constitute a homeostatic response to maintain islet beta-cell numbers in the presence of cytotoxic extracellular glucose concentrations (which is common in patients with uncontrolled Type 1 diabetes), but at the cost of reduced insulin secretion [145]. However, many other studies implicate a protective role of H_2_S in insulin signalling pathways. Studies in a mouse myoblast cell model insulin resistance reported a reduction in H_2_S production, despite elevation in CSE protein levels [146]. Treatment of these cells with exogenous H_2_S improved insulin sensitivity and mitochondrial function in part through phosphorylation and activation of the insulin receptor pathway [146]. CSE activity and H_2_S production in adipocytes also mediated translocation of glucose transporter 4 (GLUT4), an essential step in the effective uptake and utilisation of glucose [147]. Work by Xue et al. [148] showed that H_2_S donor treatment increased activation of insulin receptor and improved glucose uptake in adipocytes and myocytes and that chronic H_2_S donor treatment decreased blood glucose, improved insulin sensitivity and glucose tolerance, and elevated phosphorylation of insulin signalling pathway enzymes in a diabetic rat model. However, the beneficial effect of H_2_S donors on whole-animal carbohydrate metabolism is contradicted by Gheibi et al. [149] who showed that chronic administration of H_2_S donor compounds in a type-II diabetic rat model resulted in dose-dependent impairment of glucose tolerance, pyruvate tolerance, and insulin secretion. These two rat studies underline the importance of H_2_S donor concentration in the interpretation of the biological effects of H_2_S. The Xue et al. paper used NaSH over the range of 168–670 µg/Kg/day for 10 weeks, whereas the Gheibi et al. study used a higher range of 280–5600 µg/Kg/day for 9 weeks. The majority of the deleterious effects of chronic NaHS treatment reported by Gheibi et al. were found in the highest dosage groups, indicating that their treatment range may well approach the dosage at which NaHS begins to confer deleterious or toxic side-effects. The often contradictory work compiled to date shows that the interaction between H_2_S and the molecular, cellular, and physiological role of insulin signalling remains poorly understood. As such, any potential overlap between H_2_S and Acarbose in improving longevity and late-life health remains unresolved and more work is required to investigate this potentially important signalling commonality.

### H_2_s in lifespan shortening

#### Progeria syndromes

Progeroid syndromes are a set of genetic disorders characterised by a shortened lifespan and the development of phenotypes normally associated with advanced age [150]. Progeroid syndromes mimic many characteristics of normal human ageing to varying degrees, and therefore present invaluable insight into dysregulation of normal physiological ageing [151]. While all progeroid conditions are extremely rare, the most common is Hutchinson–Gilford progeria syndrome (HGPS). HGPS is an example of a laminopathy, a sub-set of progeria caused by various mutations in the LMNA gene which encodes for lamin proteins [150]. Lamins are a class of intermediate filaments, serving as scaffolds that anchor chromatin and transcription factors to the nuclear periphery [152]. Dysfunctional post-translational processing of lamin A leads to a permanently farnesylated and methylated lamin A isoform, named progerin. The expression of progerin produces disruption of the nuclear membrane, leading to premature senescence, and ageing. Progerin also accumulates in small amounts during physiological ageing due to spontaneous activation of the cryptic splice site observed in HGPS [153]. This suggests that normal and accelerated ageing share at least some common molecular basis. Moreover, many of the hallmarks of physiological ageing are observed in HGPS patients [154]. Overall, the link between progerin accumulation and hallmarks of ageing, the manifestation of age-related diseases in HGPS patients, the expression of progerin during normal ageing and the well-characterised genetic defects in HGPS make it a relevant human ageing model [155].

#### H_2_s in progeria

Therapeutic treatments for patients with progeroid diseases remain critically lacking, with an average life expectancy in HGPS of less than 15 years [156]. Current treatments include farnesyltransferase inhibitors, rapamycin analogues, sulforaphane, and vitamin D analogues which all have clear impacts on disease symptoms but have yet to provide substantial improvements to patient lifespan or comorbidities [156]. While no studies have investigated the role of H_2_S in HGPS to date, there is known overlap between H_2_S and the mechanisms that underpin the effects of rapamycin (see section H2S in dwarf mouse models), sulforaphane, and vitamin D treatments. Sulforaphane is an isothiocyanate compound found naturally in cruciferous vegetables that acts as a H_2_S donor. Beyond HGPS, treatment or ingestion of sulforaphane-rich vegetable homogenates is a promising treatment in Alzheimer's disease and boosts antiviral responses of natural killer cells in human clinical trials [157,158]. The mechanism through which sulforaphane operates *in vitro* appears to involve the generation of H_2_S, with sulforaphane treatment elevating H_2_S levels upon addition to cells and tissue homogenates [157,159]. Furthermore, sulforaphane treatment in a human prostate cancer cell lines impeded cancer cell survival via H_2_S-mediated JNK and MAPK signalling [159]. Finally, the activity of sulforaphane was attributed largely to its potent activation of NRF2 by modification of KEAP1 [160] and insulin signalling [161], mechanisms that are also directly influenced by H_2_S signalling (see sections Post-translational modification (persulfidation) and Acarbose and IIS signalling). Given that sulforaphane is a compound that is essentially a naturally occurring H_2_S donor and has been shown to operate through biological mechanisms that are known H_2_S signalling pathways, there have been a surprisingly limited number of studies that directly monitor H_2_S levels following sulforaphane treatment, and none in the context of HGPS. Future studies should aim to monitor H_2_S production, disposal, and activity in sulforaphane treated HGPS models to better understand the interplay between these compounds.

Vitamin D and related compounds have also been used in the treatment of HGPS [162], and while the connection to H_2_S is not as immediately evident as the H_2_S-donating sulforaphane, evidence exists for a commonality in their modes of action. Vitamin D treatment in mice elicits a dose-dependent elevation of tissue H_2_S levels in the kidney and brain [163]. Cell culture studies found that H_2_S formation was central to vitamin D-induced protection of adipocytes from inflammation and impaired glucose utilisation due to high glucose culture conditions [147]. Finally, a population study found a correlation between reduced plasma H_2_S and vitamin D levels in African American type-II diabetics compared with Caucasians with type-II diabetes, and *in vitro* studies in monocyte culture also found an elevation of CSE expression and H_2_S production following vitamin D treatment [164].

Together, the strong overlap between proven treatments for HGPS and established molecular mechanisms under the influence of H_2_S (mTOR signalling, NRF2 response, and vitamin D signalling) it is surprising there have been so few studies addressing the role of H_2_S in the management of HGPS. While there has been no research directly linking H_2_S to HGPS, there has been work published in another progeria syndrome, Werner syndrome (WS). The study showed that the cellular morphological phenotype of human WS cells, characterised by increased protein aggregation, high levels of oxidative stress and nuclear dysmorphology, was ameliorated by treatment with NaHS [165]. The beneficial effects of NaHS treatment were due to inhibition of the mTOR pathway, as rapamycin treatment displayed similar effects to NaHS treatment. Furthermore, the enzymes involved in the endogenous production of H_2_S were down-regulated in WS cells, suggesting that reduced H_2_S levels may be one of the causes of WS phenotype [165]. Overall, this study hints at the importance of the TSP and H_2_S production in WS progeria and stresses the importance of further research across all progeroid diseases.

## The potential for H_2_S therapeutics against ageing

With the accumulated evidence that H_2_S is central to physiology and pathology across species and tissues, the inevitable question is whether we can leverage our understanding of H_2_S to design translational interventions, potentially even as a treatment against ageing [166]. Studies that show clinically relevant roles for H_2_S in age-related diseases have fuelled this discussion. One such example is critical limb ischaemia (CLI), the end stage of peripheral arterial disease which is fast becoming a major morbidity in the aging population, with incidence increasing at twice the rate of global population growth and a higher global incidence than cancer, dementia, HIV/AIDs, and heart failure [167]. Islam et al. [168] examined gastrocnemius tissues sampled from post-amputation limbs of patients with CLI to interrogate regulation and signalling of H_2_S in these patients. CLI patients showed decreased transcription of CSE, CBS, and MPST mRNAs, reduced H_2_S and sulfane sulfur levels, a reduction in NRF2 and transcription of its target genes such as catalase and glutathione peroxidase and an increase in markers of oxidative stress such as malondialdehydes and protein carbonyls [168]. While their study was limited by the difficulty in obtaining human control samples from amputees without CLI, the results show a potentially pathological role of dysregulated H_2_S production and signalling in a clinical setting. Further work is required to develop this understanding and attempt H_2_S-based therapies for this growing clinical population. Another major clinical presentation in the ageing population is the increased risk of osteoporosis. A genome-wide association study (GWAS) identified nonsynonymous single nucleotide polymorphisms in the H_2_S oxidising enzyme gene SQR as a susceptibility variant in postmenopausal osteoporosis risk in Korean women [169]. Validation studies in a preosteoblast cell line found overexpression of this variant improved markers of osteoblast differentiation [169]. The study did not have a direct measure of H_2_S in individuals with this variant and so could not determine for certain if the variant resulted in an increase or decrease in the H_2_S oxidation activity of SQR. Nonetheless, this implicates H_2_S in osteoblast maintenance. This is supported by other studies that have described conflicting roles for H_2_S in bone remodelling [170,171]. Furthermore, a GWAS meta-analysis of age-related hearing impairment identified CSE as one of the seven loci that was reproducibly identified as a candidate in the onset of hearing loss [172], while another identified a variant in the promotor region of CBS in peripheral neuropathy caused by the chemotherapy treatment of multiple myeloma [173]. These studies help foster the potential for H_2_S-based therapies as they suggest a role for H_2_S in many age-related pathologies and provide novel targets for drug development.

The emerging understanding of how H_2_S exerts influence over clinically relevant biological processes raises hopes for the development of a new class of therapeutics. However, several major obstacles prevent this from being immediately achievable. The chemical nature of H_2_S itself poses the greatest challenge to its use as a therapy. The volatility of H_2_S impedes its study in basic research as H_2_S gas readily escapes into the air on the bench. Furthermore, as H_2_S reacts so readily with a wide range of other chemical species, it would prove challenging to control off-target effects in a potential H_2_S-based therapy. Of greatest concern, however, is the powerful inhibition of COX by H_2_S. It has been proposed that the regulation of H_2_S production and oxidation is so well conserved across species largely due to the necessity to precisely modulate intracellular H_2_S levels in order to avoid toxicity by COX inhibition. There may be some hope, however, that chronic administration of H_2_S need not be toxic. Reed et al. [174] investigated cognitive outcomes in the urban population of Rotorua, New Zealand where residents have been exposed to unusually high atmospheric concentrations of volcanic H_2_S for decades. As H_2_S is a known environmental toxin, their hypothesis was that this population would have reduced cognition compared with controls, but they found that areas of the city with lower (but still abnormally high) ambient H_2_S had no significant reduction in measures of cognition while those exposed to the highest levels of ambient H_2_S actually showed better performance in reaction time and in the digit symbol tests [174]. Related studies on the population of Rotorua found no association between H_2_S exposure and asthma risk, peripheral neuropathy or cancer incidence, and actually indicated a potential protective effect against Parkinson's disease [175–178]. While these studies are indicative of safe, long-term exposure to H_2_S in humans, there are limitations in their design including the difficulties in estimating the ambient H_2_S levels throughout the decades, misclassification of individuals into the wrong exposure group, and it is impossible to confirm causality for any of the observed effects as the studies were epidemiological in nature. These limitations necessitate further study to best understand the therapeutic window for safe and effective H_2_S exposure. The challenges of H_2_S therapies and the positive and negative considerations for each of the established H_2_S-donating compounds was reviewed recently [179]. Given these challenges, any progress in the development of H_2_S therapies is contingent on better measurements of tissue H_2_S concentrations *in vivo*, the improved resolution of flux through H_2_S production, oxidation, and signalling, the establishment of the therapeutic window for H_2_S compounds, and innovations in the administration and targeting of H_2_S in therapies. These are not insubstantial open questions for the field but given the rapid rise in interest of H_2_S biology in recent years, our understanding of these questions is likely to expand greatly.

## Future directions and conclusions

Increasing evidence shows that H_2_S is integral to multiple healthspan- and lifespan-extending interventions, whether dietary, pharmacological, or genetic in nature. This is due to the capability of H_2_S to participate in a multitude of biological processes by virtue of its diverse signalling modalities. There is a high degree of evolutionary conservation across taxa for the production of H_2_S itself through the transsulfuration pathway and in the signalling pathways it interacts with. Together, these attributes implicate H_2_S as a powerful modulator of healthspan, severity of disease, and longevity. However, there are many aspects of our understanding that remain vague. Most prominently, due to the short half-life and chemical promiscuity of H_2_S, it is extremely challenging to obtain accurate measures of H_2_S and related chemical species *in vivo*. This limitation means that while we are increasingly certain of a correlation between H_2_S and various markers of longevity and healthspan, it is difficult to ascertain which specific chemical species confers the observed effects and where these effects are occurring at the tissue, cellular or even sub-cellular level. In addition, while this review has focussed on the many beneficial effects of H_2_S, it should not be forgotten that excessive levels of H_2_S are extremely toxic in biological systems. As such, future research should focus on better understanding the precise mechanisms by which H_2_S operates and the development of more sophisticated methods for measuring *in vivo* H_2_S levels. Only once these advancements are made can we begin in earnest to work towards H_2_S-based therapeutics.

## Abbreviations

AKI: acute kidney injury
AMPK: AMP-activated protein kinase
CBS: cystathionine-beta-synthase
CLI: critical limb ischaemia
COX: cytochrome c oxidase
CSE, or CGL: cystathionine-gamma-lyase
CVDs: cardiovascular diseases
DR: dietary restriction
DR: dietary restriction
EAA: essential amino acids
ETHE1: ethylmalonic encephalopathy 1
GSH: glutathione
GSSH: generating glutathione persulfide
GWAS: genome-wide association study
H_2_S: hydrogen sulfide
HGPS: Hutchinson–Gilford progeria syndrome
IIS: insulin/insulin-like signalling
KO: knock-out
LTP: long-term potentiation
MPST, or TUM1: 3-mercaptopyruvate sulfurtransferase
MSBT: methylsufonylbenzothiazole
NMDA: *N*-methyl-d-aspartic acid
NMN: nicotinamide mononucleotide
NRF2: nuclear factor erythroid 2-related factor 2
PLP: pyridoxal-5′-phosphate
RAGE: receptor for advanced glycation end-products
RBCs: red blood cells
ROS: reactive oxygen species
RSS: reactive sulfur species
SAAs: sulfur-containing amino acids
sGC: soluble guanylate cyclase
SOU: sulfide oxidation unit
SQR: sulfur:quinone oxidoreductase
SRB: sulfate-reducing bacteria
TST: thiosulfate sulfurtransferase
WS: Werner syndrome
